# Knowledge mapping of exosomes in prostate cancer from 2003 to 2022: a bibliometric analysis

**DOI:** 10.1007/s12672-024-01183-x

**Published:** 2024-07-24

**Authors:** Yingjie Li, Lin Ma, Hualin Chen, Zhaoheng Jin, Wenjie Yang, Yi Qiao, Zhigang Ji, Guanghua Liu

**Affiliations:** Department of Urology, Peking Union Medical College Hospital, Chinese Academy of Medical Sciences & Peking Union Medical College, No.1 Shuaifuyuan Wangfujing Dongcheng District, Beijing, 100730 China

**Keywords:** Prostate cancer, Exosomes, Bibliometrics, CiteSpace, VOSviewer

## Abstract

**Background:**

Prostate cancer (PCa) is highly prevalent among males worldwide. The investigation of exosomes in PCa has emerged as a dynamic and important research area. To visually depict the prominent research areas and evolutionary patterns of exosomes in PCa, we performed a comprehensive analysis via bibliometric methods.

**Methods:**

Studies were retrieved from the Web of Science Core Collection. CiteSpace, VOSviewers, and the R package “bibliometrix” were employed to analyze the relationships and collaborations among countries/regions, organizations, authors, journals, references, and keywords.

**Results:**

Over the past 20 years (2003–2022), 995 literatures on exosomes in PCa have been collected. The findings indicate a consistent upward trend in annual publications with the United States being the leading contributor. *Cancers* is widely recognized as the most prominent journal in this area. In total, 5936 authors have contributed to these publications, with Alicia Llorente being the most prolific. The primary keywords associated with research hotspots include “liquid biopsy”, “identification”, “growth”, “microRNAs”, and “tumor-derived exosomes”.

**Conclusion:**

Our analysis reveals that investigating the intrinsic mechanisms of exosomes in PCa pathogenesis and exploring the potential of exosomes as biomarkers of PCa constitute the principal focal points in this domain of research.

**Supplementary Information:**

The online version contains supplementary material available at 10.1007/s12672-024-01183-x.

## Highlights

- This study is a comprehensive bibliometric analysis in the field of exosomes in PCa.
- Function and therapeutical potential serve as the foundation for this subject.
- Liquid biopsy and biomarkers would be the focus and emerging hotspots.

**Supplementary Information:**

The online version contains supplementary material available at 10.1007/s12672-024-01183-x.

## Introduction

Prostate cancer (PCa) is the predominant form of solid tumors in males, exhibiting a high mortality rate [1]. Recently, the global prevalence of PCa has risen [2]. Diagnosis of PCa typically involves digital rectal examination and assessment of serum prostate-specific antigen (PSA) levels, which serve as biological markers. Nevertheless, current diagnostic tools often lack the requisite sensitivity and specificity for the accurate detection of PCa, which poses challenges for effective management of this condition. Despite the effectiveness of androgen deprivation therapy, the current understanding of the appropriate primary approach to early-stage metastatic PCa treatment and the molecular mechanisms involved in its progression to castration-resistant PCa remain ambiguous [3]. Liquid biopsies, circulating tumor cells, exosomes, and circulating nucleic acids have been intensively studied as less invasive monitoring methods for PCa patients [4].

Exosomes are a type of extracellular vesicle with sizes ranging from 40 to 160 nm, containing numerous bioactive substances, including DNAs, RNAs, lipids, proteins, and metabolites. Almost all living cells can release exosomes, previously considered carriers or “garbage bags” responsible for eliminating cellular waste to maintain cellular homeostasis [5]. Over time, exosomes, which are abundant in diverse bodily fluids, have been recognized as multilamellar vesicles that aid in intercellular communication by transferring bioactive cargo during both physiological processes and disease states. The diverse contents of exosomes indicate their cellular origins and provide valuable information on the current state of the originating cells. Consequently, these bioactive substances hold potential as biomarkers for clinical diagnostics and the development of novel treatment strategies [6, 7].

The quantity of articles published annually on exosomes in PCa is increasing. Therefore, maintaining a consistent and comprehensive understanding of the latest advancements in this field remains a challenge. Through quantitative and qualitative measures, bibliometrics offers detailed insights into authors, keywords, journals, countries, institutions, references, and other aspects of the relevant research field [8]. Bibliometric tools such as CiteSpace [9] and VoSviewer [10] are frequently utilized to visualize the findings of analyses on publications and have been extensively used in the medical field. To the best of our knowledge, researchers have conducted bibliometric analyses on exosomes in cardiovascular diseases [5], breast cancer [11], and epithelial ovarian cancer [12]; however, the field of bibliometric studies on exosomes in prostate cancer (PCa) is still relatively young and holds vast potential for exploration. To address this knowledge gap, we aimed to analyze exosome-related publications on PCa over the past two decades (2003–2022) using bibliometrics to identify the key contributors, assess the present state of research, and predict future research trends.

## Methods

### Search strategy

**Fig. 1:**
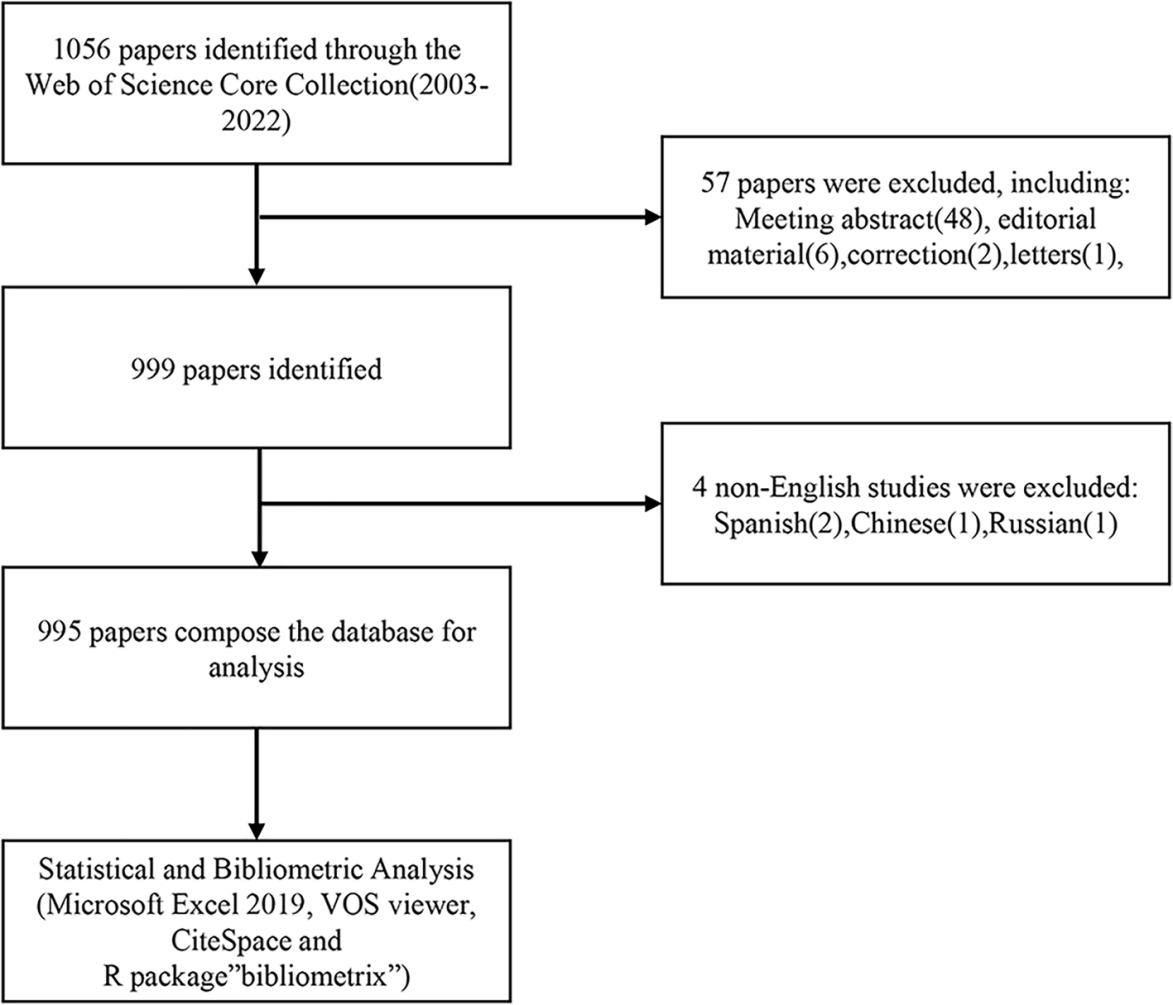
Flowchart of literature selection in this study

A literature search of the Web of Science Core Collection (WoSCC) database (https://www.webofscience.com/wos/woscc/basic-search) was conducted on July 20th, 2023. The search formula is ((TS = (Exosomes)) AND TS = (prostate cancer)) AND LA = (English), and the type of documents is set to “articles” and “review” (Fig. 1).

### Data analysis

VOSviewer (version 1.6.18), a software commonly used for bibliometric analysis, helps analyze vast amounts of research data to build networks of collaboration, co-citation, and co-occurrence [13]. The software primarily performs the following analyses: author and co-cited author, journal and co-cited journal, and cited reference co-citation networks. The VOSviewer map illustrates the nodes that symbolize various entities, including journals and authors. The node size corresponds to the quantity of items it represents, whereas the color signifies its classification. Additionally, the thickness of the lines connecting the nodes indicates the level of collaboration or co-citation among entities [14].

CiteSpace (version 6.2. R4) is bibliometric analysis software developed by Chen C [15]. In this study, CiteSpace was used to create a visualization map of country and organization analyses, keyword clustering analyses, and dual-map overlays of journals, as well as to analyze references and keywords with citation bursts.

The R package “bibliometrix” (version 4.2.2) (https://www.bibliometrix.org) served as the primary instrument to identify trend topics [16]. We also applied this method to visualize the global distribution network of exosome researches in PCa. The journal quartile and impact factor were acquired from Journal Citation Reports 2022. Additionally, a quantitative analysis of publications was performed using Microsoft Office Excel 2019.

## Results

### Analysis of annual publications

**Fig. 2:**
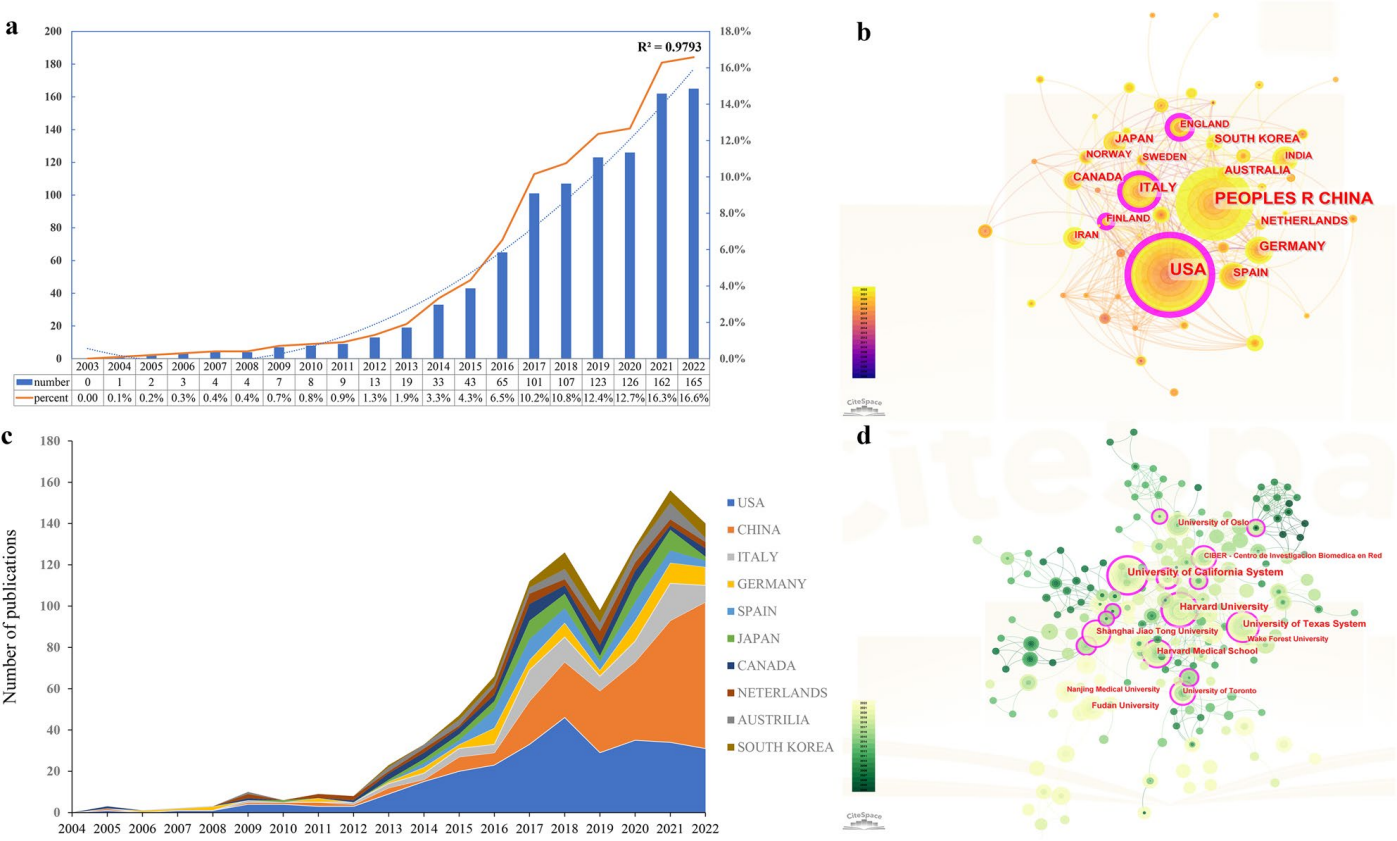
**a** Annual publication of research on exosomes in PCa. **b** The international collaboration visualization map of countries/regions. **c** The changing trend of the annual publication quantity in the top 10 countries/regions from 2003 to 2022. **d** The visualization of institutions on research of exosomes in PCa

The number of publications in a specific period is an indicator of the evolving research trends in a field (Fig. 2a). During the period 2003–2022, exosome studies on PCa showed an overall upward trend. Between 2004 and 2011, the number of articles published remained relatively low, indicating that the exploration and advancement of research on exosomes in PCa were still in their early phases. Subsequently, from 2012 to 2018, there was a consistent year-on-year rise in published papers. Notably, between 2019 and 2022, a substantial surge in the number of published articles on exosomes in PCa was expected anticipated, culminating in a total output of 165 publications in 2022. Analysis of the data revealed a statistically significant and robust relationship between the number of publications and the year of publication, as evidenced by a high R-squared value of 0.9793. Evidently, researchers have increasingly focused on the potential use of exosomes in urological tumors over time.

### Contribution of active countries/regions

We found that, in total, 995 articles were published across 65 countries/regions. The geographical distribution of these articles is visually represented in Fig. S1, which illustrates that most studies originated in North America, Europe, and Asia. Table 1 presents the top 10 countries/regions in terms of publication count, with the United States emerging as the foremost contributor, accounting for 292 papers, nearly 30% of the total publications. Figure 2b illustrates the international cooperation map created using CiteSpace, revealing the collaboration among countries. Figure 2c shows the annual number of publications from the ten most productive countries/regions, demonstrating the rapid growth of publications concerning exosomes in PCa. Analysis of collaboration networks revealed that the United States possessed the highest centrality score (0.38), indicating its position as the central hub for international collaboration in this field. Following the US were Italy (0.17) and Germany (0.07), highlighting their significant contributions to fostering global research partnerships. France is a pioneering nation in the investigation of exosomes’ role in PCa.

**Table 1:** The Top 10 countries/regions that contributed to the research of exosomes in prostate cancer

Rank	Country	Counts	Percentage	H-index	Total Citations	Average citation per paper	Centrality
1	USA	292	29.35	74	25,613	87.72	0.38
2	People R China	269	27.01	57	16,216	60.28	0.06
3	Italy	85	8.54	31	8557	100.67	0.14
4	Germany	64	6.43	31	9053	141.45	0.07
5	Spain	52	5.23	30	8589	165.17	0.06
6	Japan	51	5.13	27	8285	162.45	0.02
7	Canada	44	4.42	28	7542	171.41	0.03
8	Netherlands	39	3.92	24	7877	201.97	0.02
9	Australia	38	3.82	23	7364	193.79	0.06
10	South Korea	38	3.82	21	7222	190.05	0.02

### Contribution of high-performing organizations

A total of 1635 organizations were identified. Data presented in Table S1 indicates that the four institutions with the highest productivity are all situated within the United States. The University of California System has 35 papers, followed by Harvard University (n = 31), Harvard Medical School (n = 26), and the University of Texas System (n = 23). Total citations serve as a crucial metric for assessing the global impact of organizations. Table S1 demonstrates that the University of Oslo holds the highest number of citations, positioning it as an influential institution. The organization’s collaboration is shown in Fig. 2d through a tree-ring history visualization map. Every node on the map represents an institute, with its size reflecting the institute's publication output. The connecting lines denote the level of cooperation between organizations, with thicker lines indicating closer collaboration. The findings revealed a significant level of active cooperation among institutions, suggesting that studies conducted within these institutions may play a crucial role.

### Contribution of active authors

**Fig. 3:**
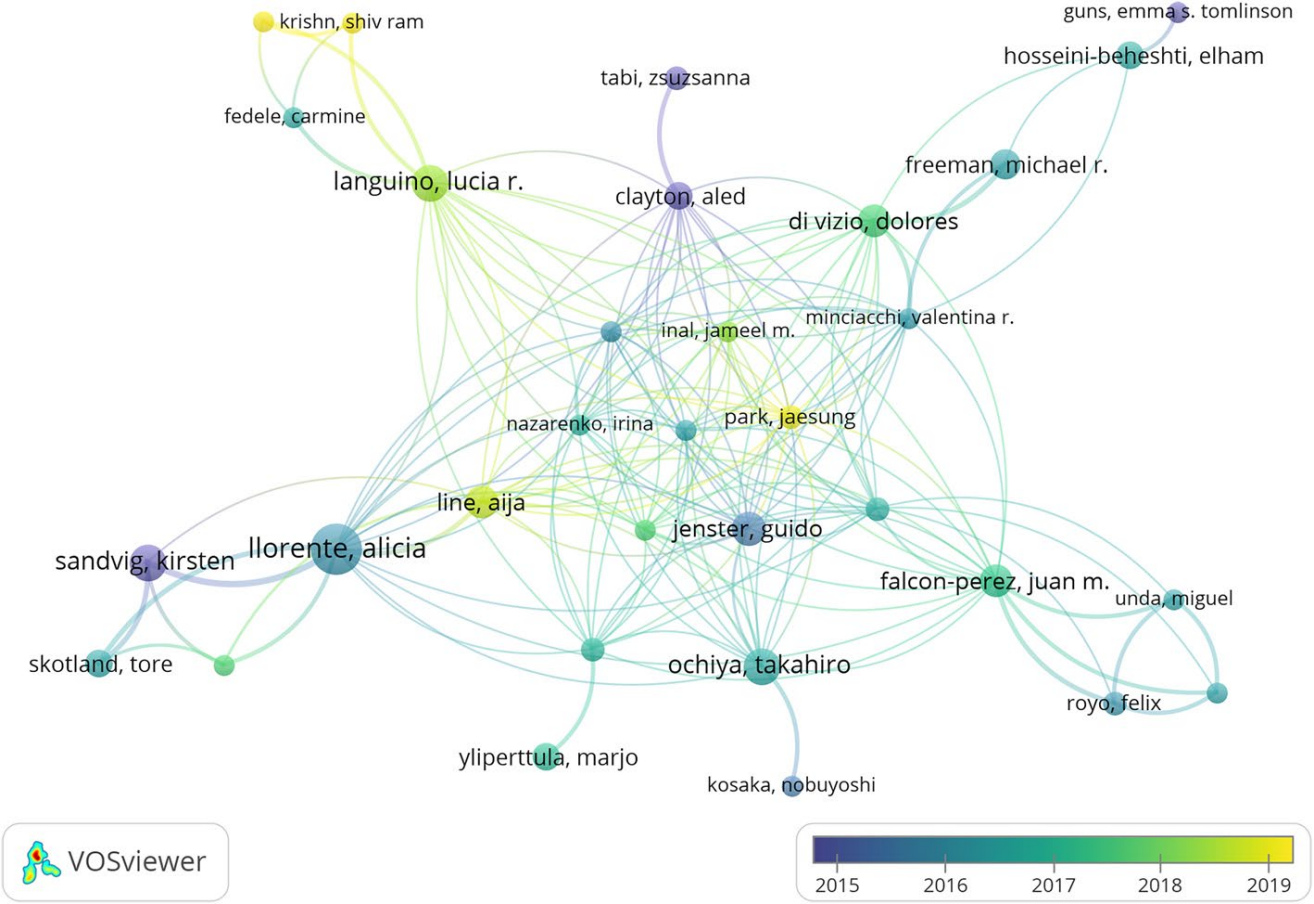
Overlay visualization map of author co-authorship analysis obtained using VOSviewer

Among the 5936 authors who have published research on exosomes in PCa, ten most prolific and co-cited authors, listed in Table S2, have significantly impacted the field and continue to drive its forward momentum. Alicia Llorente published the most papers (n = 17, 1.71%), followed by Gagan Deep (n = 13, 1.31%), Stefano Fais (n = 11, 1.11%), Mariantonia Logozzi (n = 11, 1.11%), and Kirsten Sandvig (n = 11, 1.11%). Figure 3 shows the author collaboration network, where larger nodes represent more articles published by the author. Closer cooperation among authors is symbolized by thicker lines, and we found extensive communication among authors within the field. Authors who are cited together at the same time are referred to as co-cited authors. Fig. S2 visualizes the co-cited authors exhibiting the highest total link strength. Clusters of close cooperation are indicated by different colors. Of the 554 co-cited authors, only five were cited more than 200 times.

### Analysis of influential journals and co-cited journals

**Fig. 4:**
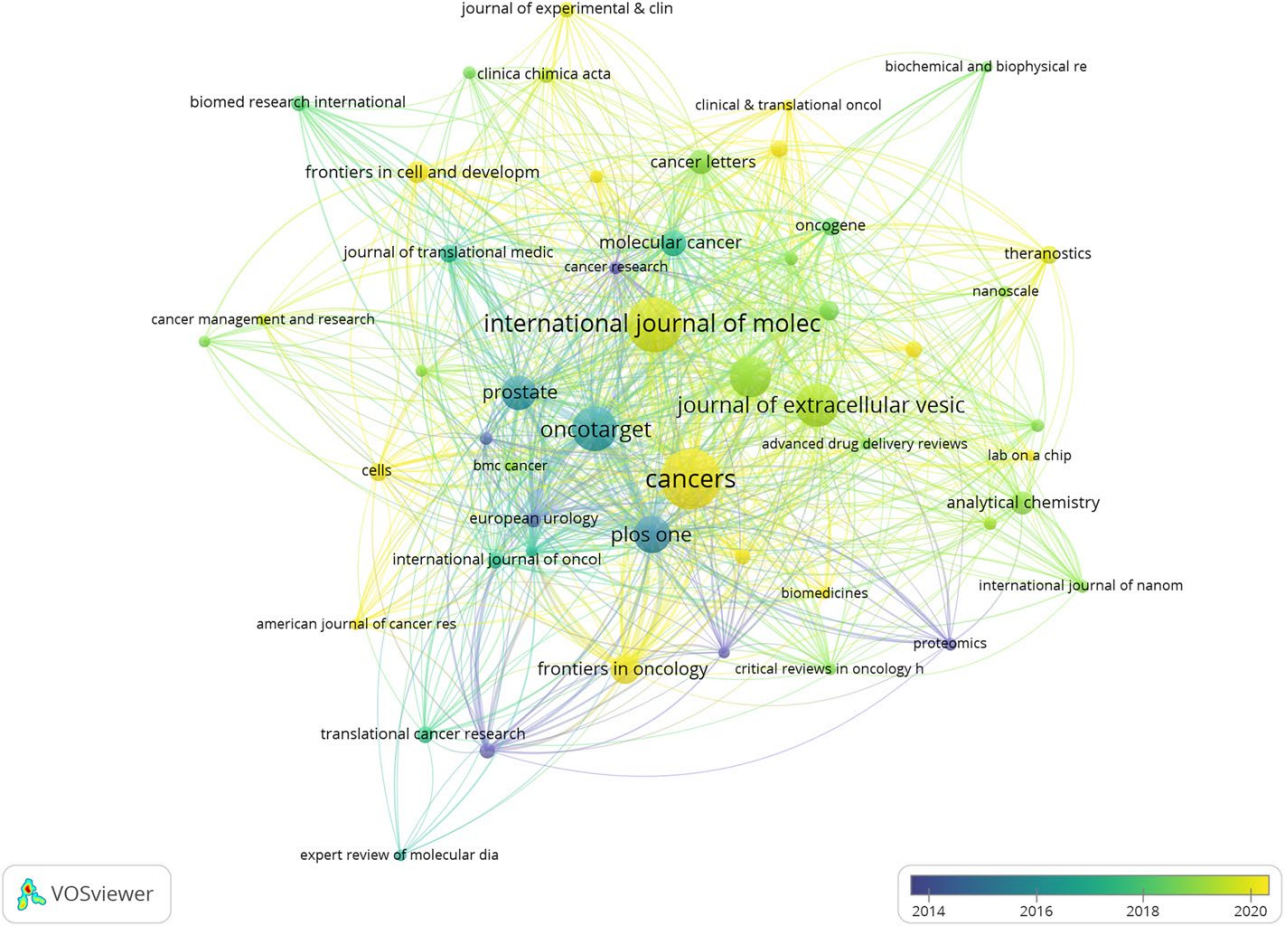
Network visualization maps of cited journals

Among all 377 journals assessed, *Cancers* (n = 40, IF = 5.2, Q2), *International Journal of Molecular Sciences* (n = 36, IF = 5.6, Q1), and *Oncotarget* (n = 28, IF = 5.168, Q1) were the top three journals in terms of publication quantity (Table S3). Furthermore, out of the top 10 most prolific journals, *Oncotarget* had the highest H-index (27), whereas *Journal of Extracellular Vesicles* exhibited the highest total citations (6237 times). Notably, the leading 10 co-cited journals, as presented in Table S3, all received over 1000 citations, with *PLOS One* being the most frequently cited (2304 citations). Figure 4 and Fig. S3 show network maps of the cited and co-cited journals created by VOSviewer. The significant overlap of journals in both maps indicates a positive citation relationship.

**Fig. 5:**
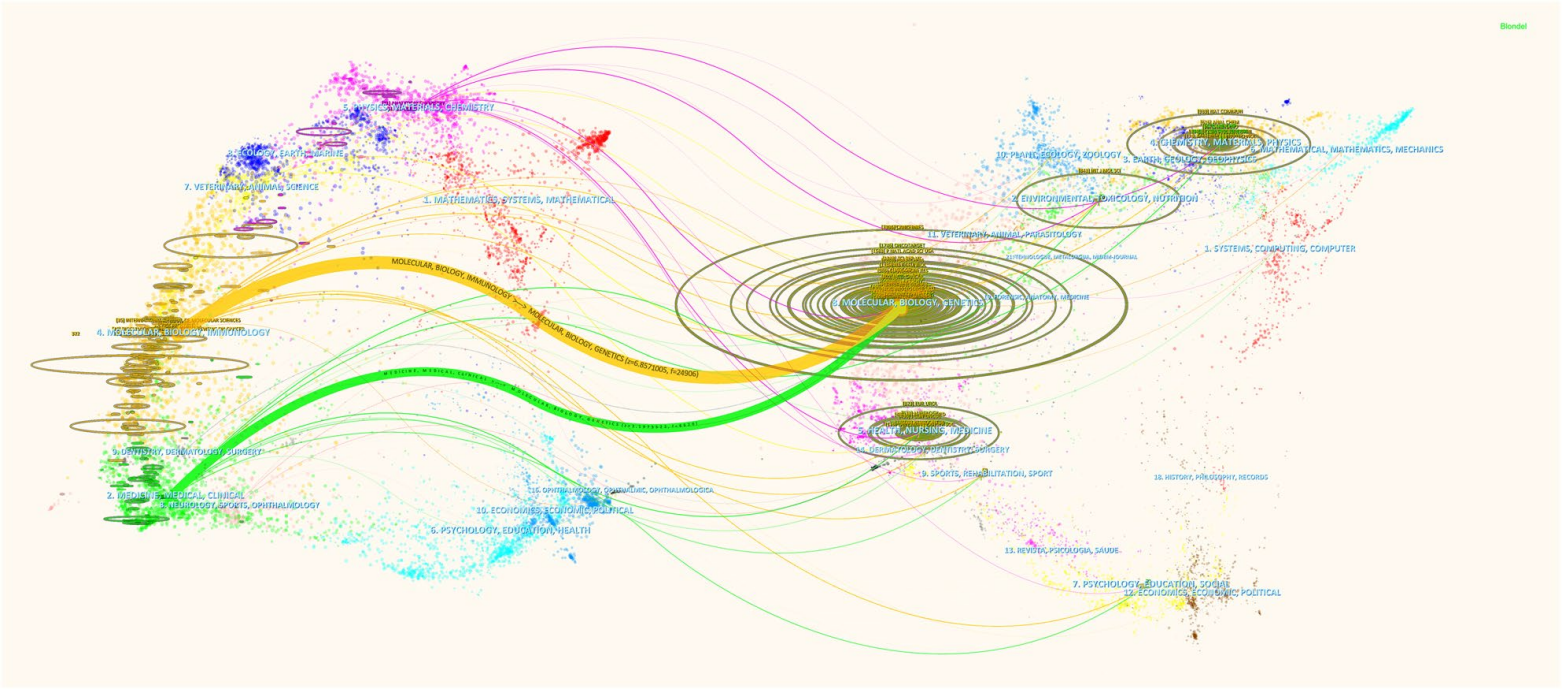
Dual-map overlap of journals obtained using CiteSpace

Using CiteSpace, a dual-map overlay was generated to visually depict the distribution of topics (Fig. 5). It’s revealed that research articles published in Molecular/Biology/Genetics and Health/Nursing/Medicine journals tended to be referenced by Molecular/Biology/Immunology or Medicine/Medical/Clinical journals.

### Analysis of references and co-cited references

The most frequently cited references are regarded as the fundamental sources of research in a particular research area. As shown in Table S4, the article “Minimal information for studies of extracellular vesicles 2018 (MISEV2018): a position statement of the International Society for Extracellular Vesicles and update of the MISEV2014 guidelines” from the *Journal of Extracellular Vesicles* by Clotilde Thery received most citations (n = 4729) [17], followed by “Secretory mechanisms and intercellular transfer of microRNAs in living cells” (n = 1457) [18] and “Current knowledge on exosome biogenesis and release” (n = 1327) [19].

**Fig. 6:**
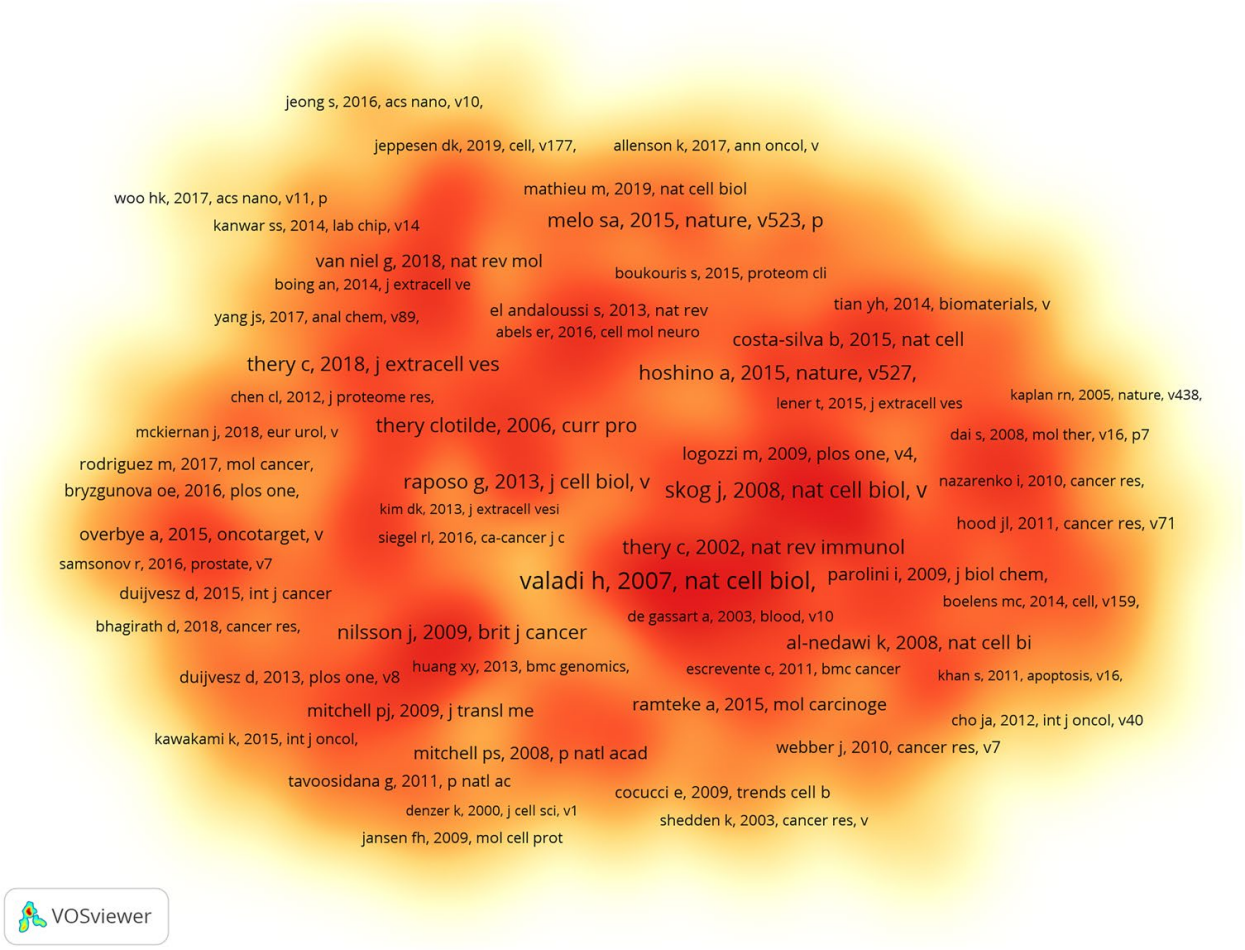
Spectral density map of the co-citation reference was obtained with VOSviewer

To provide a better overview of the research on exosomes in PCa, we compiled a list of the 10 most frequently cited references in Table S5 and constructed the co-citation density map in Fig. 6. Among all cited references, 14 had citations exceeding 100 times, whereas the top three references were cited over 150 times each. The most frequently cited reference was titled “Exosome-mediated transfer of mRNAs and microRNAs is a novel mechanism of genetic exchange between cells”, demonstrating exosomes as critical factors for intercellular communication.

References with citation bursts refer to those frequently cited by scholars during a given timeframe in each field. Fig. S4 displays the top ten references exhibiting the strongest citation bursts. The initial citation burst can be traced back to 2008. In general, the burst strength of these 10 references varied between 3.38 and 55.11, while the durability ranged from 2 to 4 years.

### Analysis of keyword co-occurrence

By conducting a co-occurrence analysis of keywords, prominent areas of specific research can be efficiently identified. Table 2 shows the 20 most frequent keywords used in the research on exosomes in PCa. Certain keywords like “exosomes”, “extracellular vesicles”, “prostate cancer”, and “cells” have been excluded. Instead, the table predominantly comprised keywords such as “expression”, “biomarkers”, “liquid biopsy”, “identification”, “growth”, “progression”, “microRNAs”, and “metastasis”. The keywords “biomarkers” and “liquid biopsy” were mentioned more than 80 times each, indicating that they represent the primary research focus within the study of exosomes in PCa.

**Table 2:** Top 20 keywords on research of exosomes in prostate cancer

Rank	Keyword	Count	Centrality	Rank	Keyword	Count	Centrality
1	prostate cancer	592	0.32	11	growth	51	0.02
2	extracellular vesicles	342	0.01	12	progression	48	0.03
3	exosomes	265	0.35	13	microRNAs	35	0.03
4	expression	209	0.14	14	metastasis	29	0.01
5	microvesicles	134	0.07	15	tumor derived exosomes	22	0.03
6	cells	129	0.07	16	proliferation	20	0.01
7	biomarkers	128	0.05	17	membrane vesicles	17	0.14
8	breast cancer	96	0.06	18	proteomic analysis	12	0.08
9	liquid biopsy	80	0.01	19	colorectal cancer	11	0.07
10	identification	57	0.07	20	circulating microRNAs	11	0.00

**Fig. 7:**
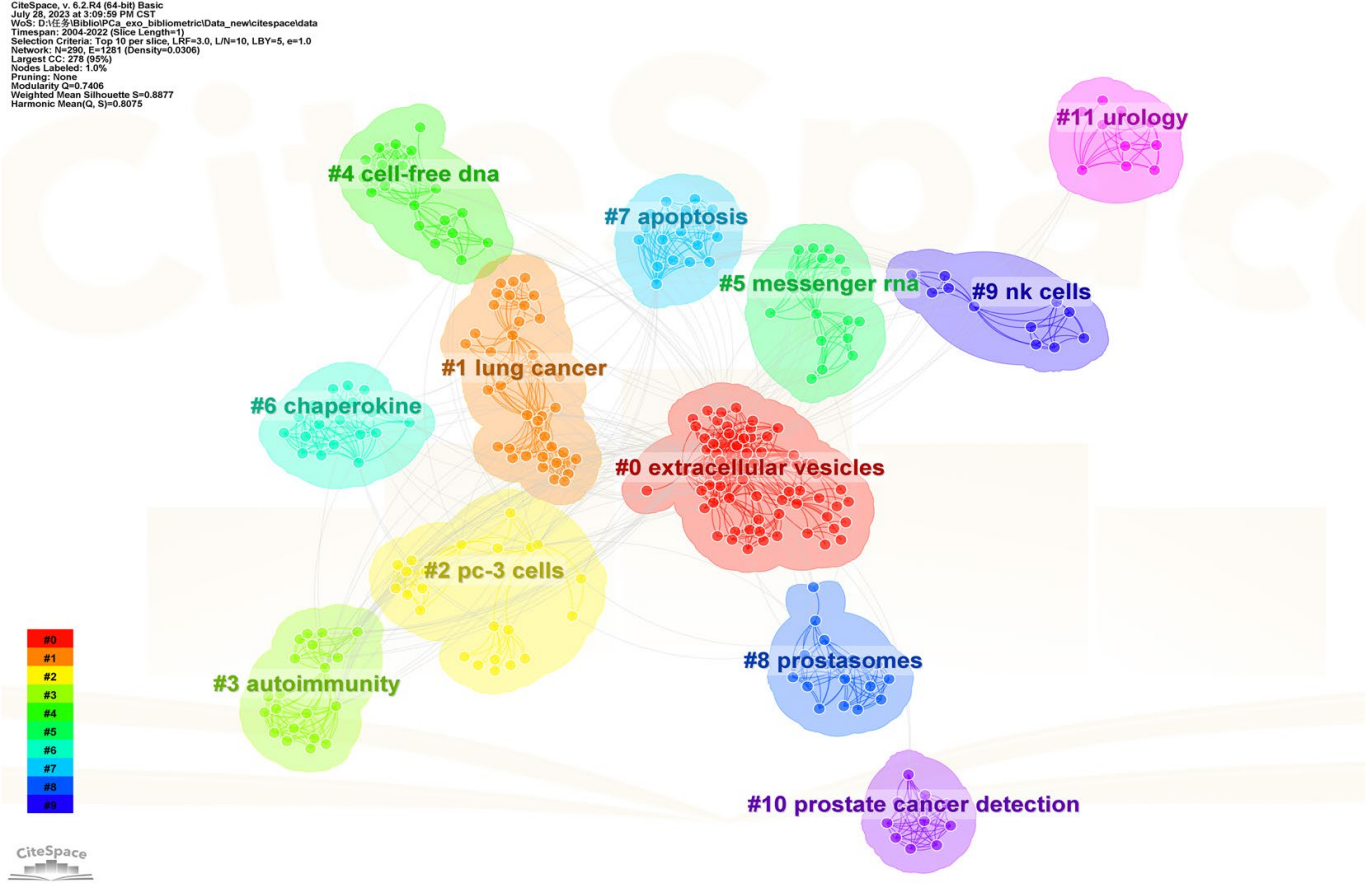
Cluster view map of keyword occurrence analysis

The CiteSpace clustering program was employed to perform clustering analysis on co-occurring keywords, thereby discerning the prevalent subjects of the interconnected literature. Figure 7 shows that 12 clusters were identified, each consisting of multiple closely-associated terms. The Q value (Q-value) was 0.7406 and the mean silhouette value (S-value) was 0.8075, indicating a substantial and compelling clustering structure.

**Fig. 8:**
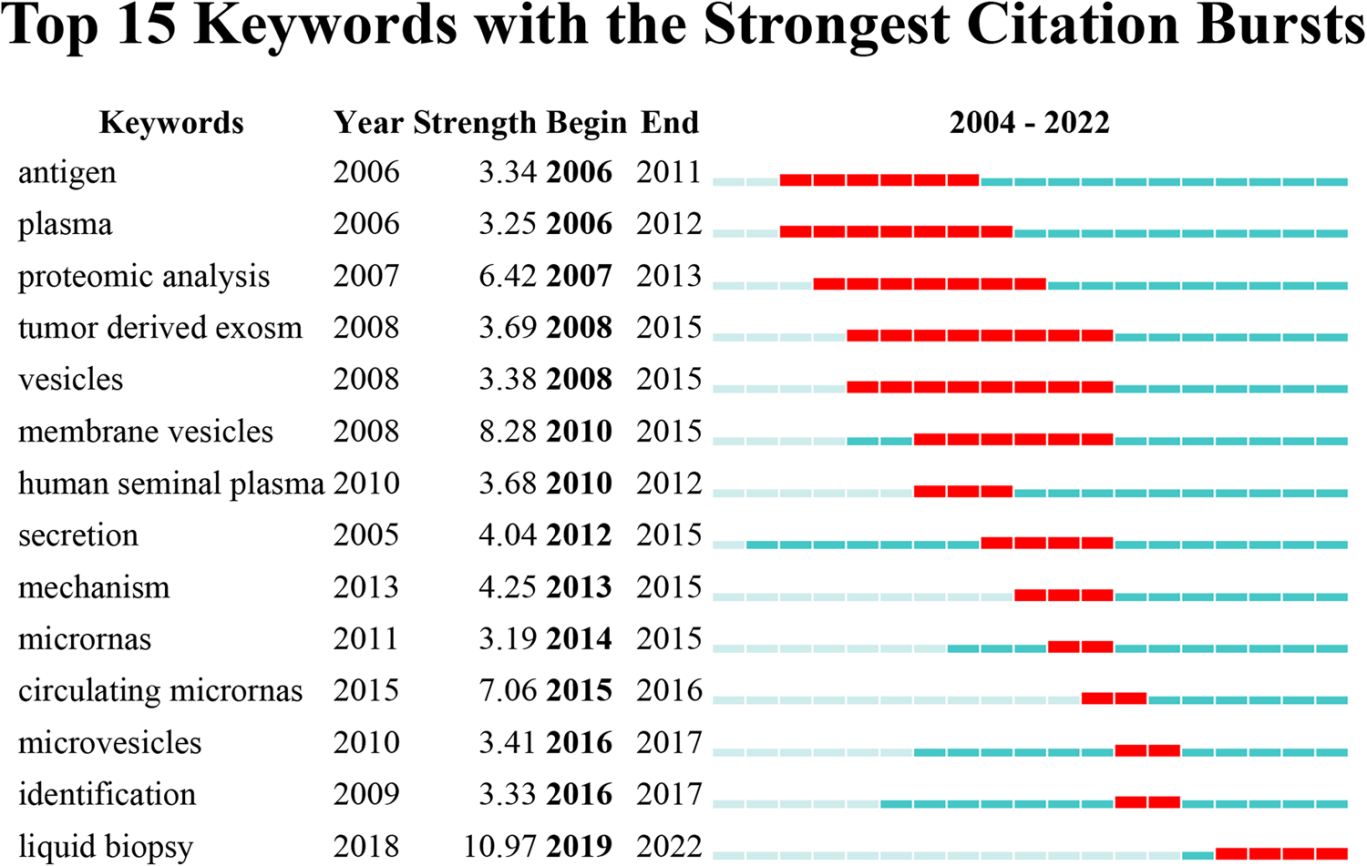
Top 15 keywords with the highest citation bursts generated by CiteSpace software

Figure 8 depicted the top 15 keywords with the strongest citation bursts, as identified by CiteSpace. Combining high-frequency keywords in Table 2, those related to exosomes in PCa with lasting citation bursts until 2022 were as follows: “liquid biopsy”, “identification”, “growth”, “microRNAs”, “tumor-derived exosomes”, “membrane vesicles”, and “proteomic analysis”. These keywords were of particular interest in our study because of their significant impact on delineating the forefront of PCa exosome research.

**Fig. 9:**
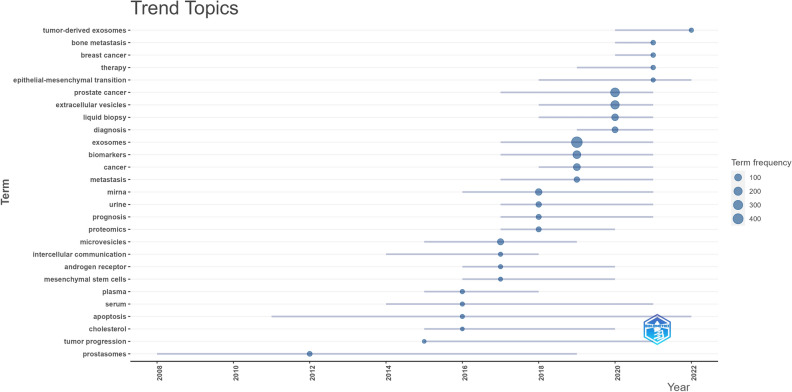
Trend topic analysis using R package “bibliometrix”

Based on the mutual interaction between keywords, the trend topic contributes to the exploration of the evolving landscape and key stages of a particular field. Figure 9 presents a trend topic analysis of exosomes in PCa, generated using the R package “bibliometrix”. In 2004–2015, this research topic did not receive significant attention, with the primary focus being on “prostasomes” and “tumor progression”. However, from 2015 to 2019, a notable increase in research on exosomes in PCa was observed, accompanied by an acceleration in related mechanistic studies. The main keywords were “liquid biopsy”, “diagnosis”, “biomarkers”, “metastasis”, “miRNA”, “urine”, “prognosis”, “proteomics”, “androgen receptor”, and “apoptosis”. Over the past four years, researchers have investigated the possible benefits and significance of exosomes in medical treatment. The main keywords were “tumor-derived exosomes”, “bone metastasis”, “therapy”, and “epithelial-mesenchymal transition”.

## Discussion

### General information

Unlike reviews or meta-analyses, a bibliometric analysis provides a thorough overview of the progression of the study field and identifies the areas of research emphasis. Although Zhu et al. explored research related to prostate cancer and exosomes from 2010 to 2022 [20], this study provides more data and perspectives, offering a more comprehensive understanding of the field.

PCa is a prevalent form of malignancy in males, and exosomes have been reported to exert an important effect on PCa proliferation, invasion, and metastasis. As shown in Fig. 2a, the enthusiasm for studying exosomes in PCa has increased steadily each year. Nevertheless, when it comes to countries/regions involved, limited participation and publication output were observed, with less than half exceeding 10 articles. The United States, with remarkable number of publications and outstanding H-index, was the leading contributor in this field. Notably, although China has published more than 250 papers in this field, its average citations per paper fell behind the other top 10 nations. Therefore, directing attention toward two key areas is imperative for altering the existing state of affairs: first, enhancing collaboration and fostering interaction with other nations, particularly the United States, Italy, and Germany; and second, closely monitoring scientific advancements to enhance the standards of researches.

The location of the top four productive organizations, all being in the United States, may be a contributing factor to their substantial impact on the field of exosome research in PCa. Figure 2d reveals that, although organizations have established cooperation networks, the scope and intensity of collaboration are suboptimal, as indicated in Table S1. Research collaborations at institutions tend to be primarily internal, with few initiatives fostering cross-border partnerships and knowledge sharing. This prevailing circumstance hinders the advancement of the research domain. Therefore, research institutions across various nations should collaborate and communicate to eliminate academic obstacles and foster scientific research.

In terms of author analysis, Alicia Llorente, a scholar from Switzerland, topped the list of contributors with 17 articles. Llorente et al. studied exosomes, biomarkers, and lipids [21]. This group conducted a well-known study in which they employed an advanced shotgun and targeted molecular lipidomic assays to analyze the lipidomes of exosomes released by the metastatic PCa cell line, PC-3. Their findings revealed a significant abundance of glycosphingolipids, sphingomyelin, cholesterol, and phosphatidylserine in exosomes derived from PC-3 cells, enhancing our understanding of the mechanisms underlying exosome formation, release, and function [22]. Furthermore, certain lipids enriched in exosomes are potential cancer biomarkers. In a subsequent investigation conducted by Llorente in 2017, significant differences in the quantities of nine lipid species were observed between prostate cancer patients and healthy controls [23]. This is the first study to demonstrate the potential use of urine exosomal lipids as PCa biomarkers. Clotilde Thery was the most frequently co-cited author, with Hadi Valadi, Héctor Peinado, and Graça Raposo following behind. It is worth noting that, despite the substantial quantity of publications and citations attributed to both contributing and co-cited authors, their impact on this subject remains inadequate. Consequently, prioritizing the volume of publications and enhancing the significance of the findings in future studies is imperative.

Regarding the journal analysis, most research on exosomes in PCa was published in *Cancers*, indicating it is currently the most extensively recognized and impactful journal in this research field. Among these journals, the journal with the highest impact factor is *Molecular Cancer* (IF = 37.3), followed by the *Journal of Extracellular Vesicles* (IF = 16). Furthermore, the analysis of co-cited journals revealed that the majority of them are regarded as Q1 journals with significant impact factors. Hence, the investigation of exosomes in PCa represents a field of active research. Notably, the current research on exosomes in PCa predominantly appears in journals confined to molecular biology and immunology, with limited representation in clinically-oriented journals. The future will witness a shift towards clinical translation of this research.

### Knowledge base

A co-cited reference is one that has been cited in multiple publications, indicating its significance as a research foundation in a particular field. This study utilized bibliometric method to identify the top ten co-cited sources in PCa exosome research. These sources serve as the foundation for studying exosomes in PCa.

Valadi et al. published the most co-cited and earliest article among the ten most-cited references in 2007. This study highlighted the ability of exosomes to facilitate intercellular communication by transmitting mRNAs and miRNAs, thus establishing a basis for further investigation of the mechanisms of exosomes [24]. Currently, miRNAs are a prominent research area in the context of PCa exosomes. In the following year, Professor Johan Skog published an article showing that exosomes released by glioblastomas containing RNAs and proteins could promote tumor behavior, and these tumor-derived exosomes may offer diagnostic insights and contribute to cancer therapy [25]. Moreover, in 2012, Professor Héctor Peinado revealed that exosomes derived from melanoma cells play a supportive role in the progression and spread of tumors. This effect is primarily achieved by instructing bone marrow progenitor cells to adopt pro-vasculogenic and pro-metastatic characteristics through upregulation of the oncoprotein MET [26]. In 2015, Hoshino published the 5th most co-cited paper in Nature, which focused on the role of exosomes in PCa metastasis. Distinct patterns of integrin expression have been revealed using exosome proteomics, and the presence of integrins within exosomes determines organotropic metastasis [27]. This study demonstrated that exosomes derived from tumors are internalized by organ-specific cells by carrying specific contents, thereby facilitating pre-metastatic niche formation.

In addition to functional studies, Raposo published a comprehensive review summarizing advancements in exosome formation, targeting, and function [28]. Similarly, Colombo published a review in the subsequent year focusing on the biogenesis and interaction of exosomes with other extracellular vesicles, particularly emphasizing the definition [29]. In 2009, Nilsson described a novel approach using urine exosomes as PCa biomarkers [30]. In 2015, Melo et al. conducted a study indicating that Glypican-1 enriched in cancer cell-derived exosomes could be used to detect early pancreatic cancer [31].

Collectively, the top 10 co-cited references primarily addressed the components of exosomes, the role of exosomes in tumorigenesis and metastasis, and their prospective utilization as diagnostic biomarkers, thereby establishing a foundational research framework in the field of exosomes in PCa.

### Emerging hotspots

Detecting reference citation bursts involves identifying references that have been frequently cited for a specific period. In our study, CiteSpace identified the top 10 references with significant citation bursts. As shown in Fig. S4, the reference with the strongest citation bursts was an extracellular vesicles study guideline. The article titled “Melanoma exosomes educate bone marrow progenitor cells toward a pro-metastatic phenotype through MET” had the second-highest citation bursts, lasting from 2014 to 2017, and was published in Nature Medicine by Héctor Peinado. It is also among the top 10 co-cited references and has been discussed in the preceding paragraph. Based on prominent citations in relevant references, the current focus of exosome research in PCa involves investigating the biological function and pathogenesis of endogenous exosomes, as well as exploring the potential of exosomes as clinical biomarkers.

Keywords, along with references with citation bursts, can expedite the rapid comprehension of the dissemination and progression of prominent areas of study within exosomes in PCa. By combining Figs. 8 and 9, it becomes apparent that the bursting keywords align with the emerging hotspots mentioned before. Among keywords with citation bursts, such as “growth”, “circulating microRNAs”, “proteomic analysis”, and “mechanism”, research on the pathological mechanism of exosomes in PCa will continue to be the central hotspot of this field in the future. A cluster analysis of keywords further substantiates this conclusion. It is worth mentioning that “liquid biopsy” and “identification” were also bursting keywords, suggesting that more relevant research will be performed in the future. Moreover, a comprehensive and in-depth investigation of their functional mechanism can potentially enhance their therapeutic potential for clinical applications. Based on the aforementioned analyses, it can be inferred that the primary areas of focus in PCa research pertaining to exosomes are as follows:

### Function and therapeutic potential of exosomes in PCa

Exosomes contain proteins, nucleic acids, lipids, and other components, and their specific composition depends on the source cells [32]. Proteins are the main components of exosomes and actively participate in several processes, such as exosome structure formation, membrane transport and fusion, antigen presentation, and tumorigenesis [33]. Exosomes carry numerous nucleic acids, and their lipid bilayer structures protect the nucleic acids from degradation. In addition, the presence of sphingomyelin, cholesterol, saturated fatty acids, and other constituents within exosomes is strongly correlated with the abundance of lipid-raft-associated and anchoring proteins [34].

Exosomes play an important role in tumor behavior, particularly in PCa progression [35]. They contribute to various processes, including the promotion of epithelial-mesenchymal transition and angiogenesis, thereby facilitating tumor invasion, creating a conducive microenvironment for tumor growth and distant metastasis, and aiding in tumor immune evasion [2, 36, 37]. Furthermore, exosome-mediated interactions between tumor cells and components of the extracellular matrix, such as cancer-associated fibroblasts and macrophages, contribute to the proliferation of tumor stem cells [38].

For example, castration-resistant prostate cancer cells secrete exosomes containing integrin alpha-2, which enhance focal adhesion kinase and extracellular signal-regulated kinase 1 and 2 activity in AR-positive cells to trigger epithelial-mesenchymal transition, ultimately facilitating the proliferation, migration, and invasion of recipient cells [39]. In terms of tumor immune evasion, exosomes derived from PCa cells selectively decrease the level of natural killer group 2, member D (NKG2D), on natural killer and CD8 + T cells by expressing NKG2D ligands on their surfaces, impairing the killing effect of these cytotoxic cells and promoting PCa cell immune escape [40]. To determine the miRNA content that could induce distinct effects on the tumor microenvironment, Sánchez et al. analyzed the differential expression of miRNAs in the exosomes of bulk and cancer stem cells using next-generation sequencing. miR-21, miR-30, and miR-218 participate in the preparation of the pre-metastatic niche by regulating osteoblast differentiation and upregulating receptor activator of nuclear factor kappa-b ligand [41]. In addition to proteins and nucleic acids, alterations in the lipidomic profile of exosomes also exert an impact on their progression [42]. Considering the significant role played by differentially expressed components within exosomes in tumorigenesis, progression, metastasis, and the overall pathological processes of PCa, targeted therapeutic interventions toward this aspect could potentially enhance clinical treatment strategies, either alone or in combination with other therapeutic modalities. Sirtuin 6 (SIRT6) has been found to be correlated with PCa progression via activation of the Notch pathway. The therapeutic effect of silencing SIRT6 has been tested in xenograft mouse models using aptamer-modified exosomes carrying small interfering RNA [43]. Nevertheless, although great advances have been made in revealing the crucial role of exosomes in PCa, additional investigations are needed to explore the underlying mechanisms in greater detail.

The active function of exosomes in various PCa biological and pathological processes, as well as their special structure, make them a potential therapeutic tool for tumors, which has aroused a passion for research. Extensive efforts have been made to explore the medical applications of exosomes, including their use as drug delivery vehicles, vaccination agents, and cell-based therapies [42]. Exosomes possess improved stability in circulation and the ability to permeate biobarriers. As a result, they can be utilized to enhance target organ regulation, leading to the efficient eradication of tumor cells while reducing drug-related side effects [4]. In a cellular experiment conducted by Saari et al., exosomes secreted by PCa cells were used to deliver paclitaxel to their parental cells, resulting in enhanced chemotherapy cytotoxicity [44]. Recent research has focused on engineering exosomes to manipulate specific molecules to affect physiological behaviors, including cargo loading, purification, and isolation. One notable example involves the modification of exosomal membrane lipids through two promising strategies: decoration of natural extracellular vesicles with lipid moieties [45] and the utilization of bioinspired and hybrid liposome-extracellular vesicle engineering approaches [46].

Although various studies have illustrated the function and components of exosomes in the pathological progression of PCa, the specific mechanisms and developing new targets for PCa therapy are still worth exploring in the future.

### Liquid biopsy and biomarkers

The conventional approach for diagnosing PCa involves evaluating PSA levels in the blood, conducting digital rectal examinations, and performing biopsies guided by transrectal ultrasonography. PSA is generated by prostate cells and not exclusively by cancer cells. Consequently, the limited specificity of this marker often results in overdiagnosis and overtreatment. Therefore, it is critically important to enhance existing processes by exploring noninvasive, cost-effective, and highly accurate biomarkers [47]. Liquid biopsies can be categorized into three primary groups depending on the origin of the tumor-derived materials discovered in the biofluids: circulating tumor DNA, circulating tumor cells, and tumor-derived exosomes, along with other extracellular vehicles [48]. Owing to their lipid bilayer structure, exosomes exhibit remarkable stability in biofluids such as plasma and urine, enabling their isolation for clinical assessment even during the initial phases of the disease [49].

Over the past few years, extensive research has been conducted on exosomes containing miRNAs to explore their potential as PCa biomarkers. miRNAs are functionally linked to tumor behavior and encompass activities such as growth, angiogenesis, metastasis, and apoptosis [50]. Various types of exosomal miRNAs, which hold promise as diagnostic biomarkers for early-stage PCa, have been detected in the blood and urine samples of PCa patients [51]. Furthermore, the amalgamation of multiple exosomal miRNAs or utilization of the proportion between one miRNA and another frequently leads to a more specific and precise PCa diagnosis [52, 53]. Several exosomal proteins have been clinically studied as potential cancer biomarkers. In a prospective study using nanoparticle tracking analysis, immunocapture-based ELISA, and nanoscale flow cytometry, circulating exosomes expressing both CD81 and PSA were found to be promising tools for differentiating PCa patients from healthy individuals or benign prostatic hyperplasia patients, with an impressive specificity and sensitivity rate of nearly 100% [54].

Noninvasive collection of urine allows for the retrieval of fluid expelled from the prostate gland, which may better reflect the disease condition. The use of urine exosomes as noninvasive biomarkers may have advantages in diagnosing, prognosticating, and monitoring PCa.^48^ The ExoDx Prostate (IntelliScore) (EPI, Exosome Diagnostics, Waltham, MA, USA) was used to evaluate the exosomal RNA levels of ERG, PCA3, and SAM Pointed Domain ETS Transcription Factor, which are involved in PCa progression. This assessment has been successfully validated in more than 1000 patients through two prospective validation trials to discriminate cancer from benign diseases [55, 56]. Further research has unraveled a proteomic map of exosomes in PCa by density-based fractionation of urine, providing more information on exosomal biomarker discovery in PCa. In addition to nucleic acids and proteins, lipids and glycans are recognized as potential biomarkers [22, 57]. Furthermore, a surge in curiosity surrounds alternative origins of exosomal biomarkers, particularly semen, which is a promising direction for further exploration.

Our study had several advantages. First, we employed widely used bibliometric tools concurrently for our investigation, ensuring a highly objective data analysis process. Second, bibliometric analysis surpasses traditional reviews by providing a broader understanding of current hotspots and frontiers in the discipline.

This study is subject to certain limitations. Notably, its reliance solely on WoSCC data excludes potentially relevant research published in other databases. Second, our inclusion criteria involved filtering studies published exclusively in English, potentially leading to an underestimation of papers written in non-English. Additionally, inadequate data precluded the incorporation publications from the year 2023 in this study.

## Conclusion

The field of exosome research in PCa has experienced remarkable advancement, evidenced by a surge in annual publications. The United States has emerged as a frontrunner country in terms of productivity, with numerous prolific authors and affiliations contributing to the field. However, increased global collaboration on exosome research is needed. Notably, keyword networks analysis reveals a primarily clinical focus in the field, emphasizing the exploration of exosome applications in PCa diagnosis and personalized therapy. In this study, we provided a comprehensive understanding of exosomes in PCa, encompassing their function, therapeutic potential, and their role in liquid biopsy and biomarker discovery. These emerging areas offer promising opportunities for future research.

### Supplementary Information

Additional file 1.

## Data Availability

The original data presented in the study are included in the article and/or Additional File. Further inquiries regarding the raw data can be directed to the corresponding author.

## Abbreviations

PCa: Prostate cancer
PSA: Prostate-specific antigen
WoSCC: Web of Science Core Collection
SIRT6: Sirtuin 6
NKG2D: Natural killer group 2, member D
