# Supplementary material for: Knowledge mapping of exosomes in prostate cancer from 2003 to 2022: a bibliometric analysis
Source: Discov Oncol. 2024 Jul 24;15:307. doi: 10.1007/s12672-024-01183-x (PMC11269540; doi:10.1007/s12672-024-01183-x)
Supplement: Supplementary file 1 — Additional file 1. [file 12672_2024_1183_MOESM1_ESM.pdf]

Supplementary Materials: Knowledge mapping of exosomes in prostate cancer in the past twenty years: A bibliometric analysis

Yingjie Li[1], Lin Ma[1], Hualin Chen[1], Zhaoheng Jin[1], Wenjie Yang[1], Yi Qiao[1], Zhigang Ji[1], Guanghua Liu[1*]

[1]Department of Urology, Peking Union Medical College Hospital, Chinese Academy of Medical Sciences & Peking Union Medical College, Beijing, China

***Correspondence:**

Guanghua Liu

pumch_liuguanghua@outlook.com

No.1 Shuaifuyuan Wangfujing Dongcheng District, Beijing, 100730, China.

List：

Supplementary	Description
**Fig. S1**	Geographic distribution map based on the total publications of different countries/regions
**Fig. S2**	Network visualization map of co-cited authors obtained on VOSviewer
**Fig. S3**	Network visualization map of co-cited journals was produced by VOSviewer
**Fig. S4**	Top 10 references with the strongest citation bursts generated by CiteSpace software
**Table S1**	Top 10 affiliations that contributed to the research of exosomes in prostate cancer
**Table S2**	Top 10 authors and co-cited authors related to exosomes in prostate cancer
**Table S3**	Top 10 journals and co-cited journals related to exosomes in prostate cancer
**Table S4**	Top 10 cited papers concerning the research of exosomes in prostate cancer
**Table S5**	Top 10 co-cited references on research of exosomes in prostate cancer

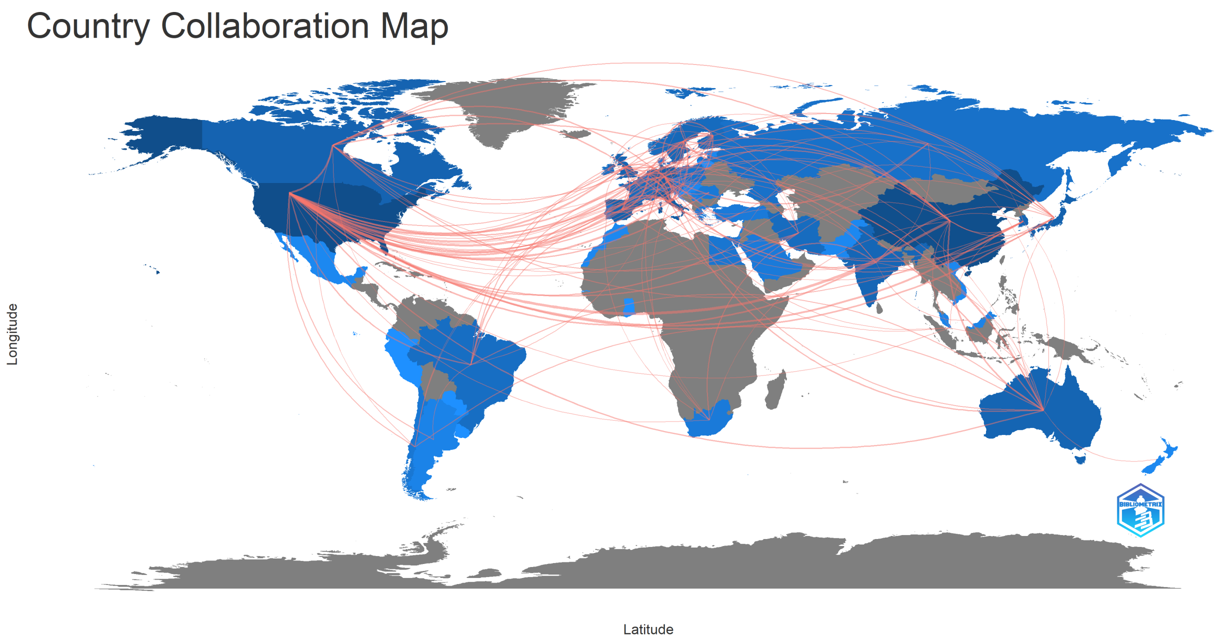

Fig. S1 Geographic distribution map based on the total publications of different countries/regions

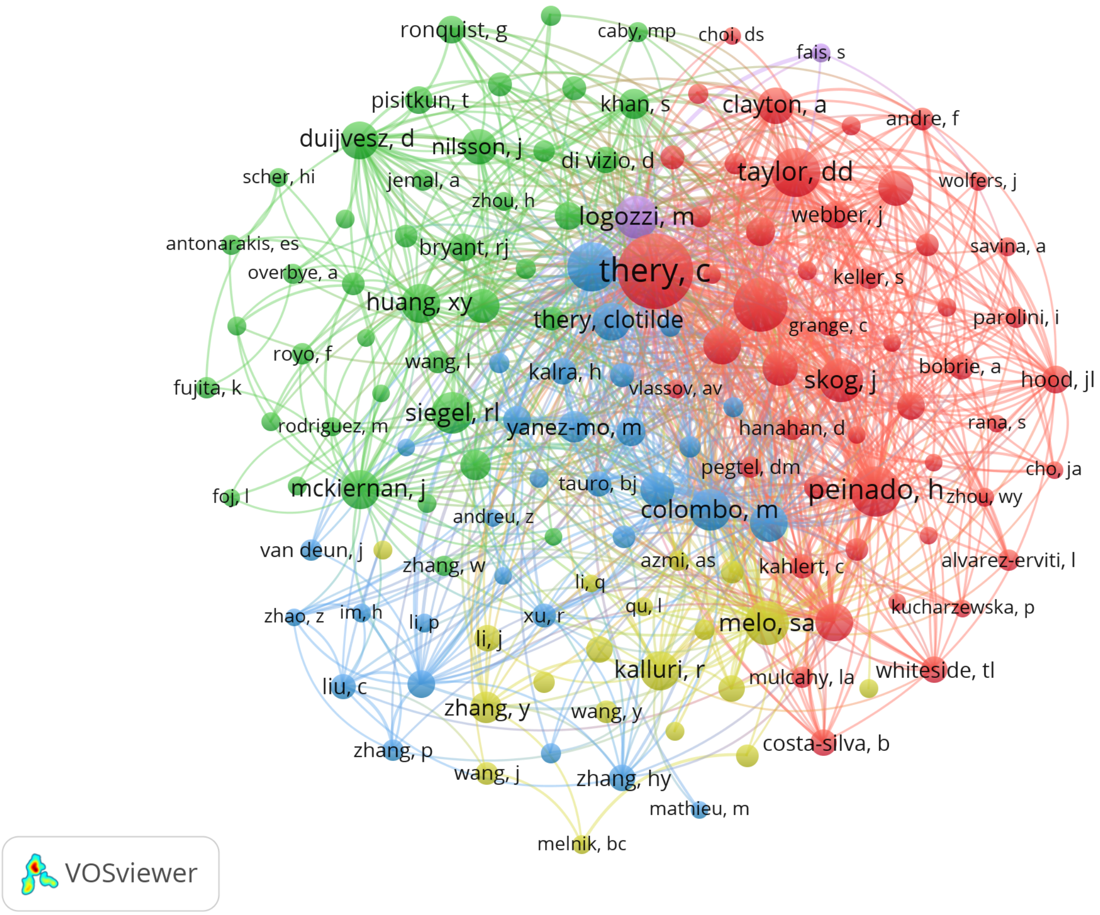

Fig. S2 Network visualization map of co-cited authors obtained on VOSviewer

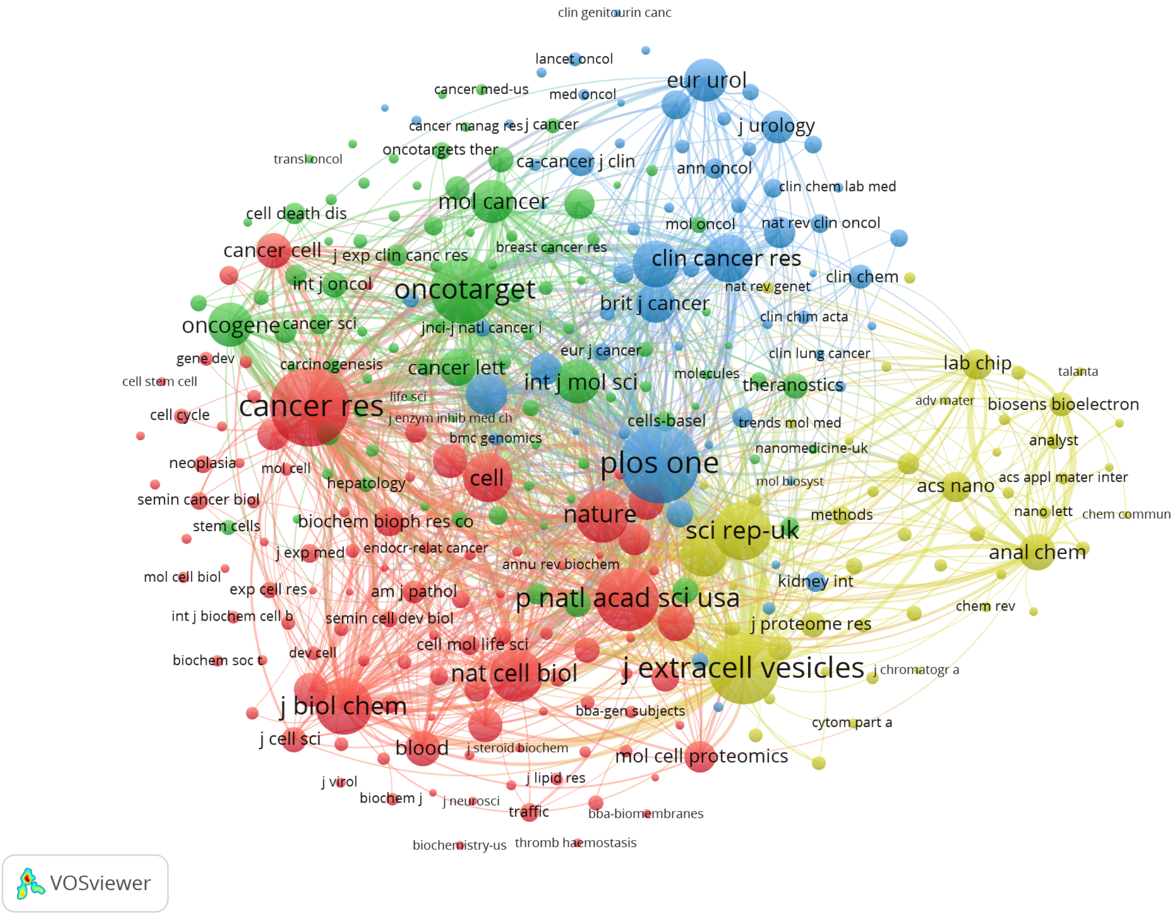

Fig. S3 Network visualization map of co-cited journals was produced by VOSviewer

Top 10 References with the Strongest Citation Bursts

References	Year	Strength	Begin	End	2004 - 2022
Yu X, 2006, CANCER RES, V66, P4795, DOI 10.1158/0008-5472.CAN-05-4579, DOI	2006	3.38	**2008**	2009	
Valadi H, 2007, NAT CELL BIOL, V9, P654, DOI 10.1038/ncb1596, DOI	2007	7.03	**2009**	2012	
Skog J, 2008, NAT CELL BIOL, V10, P1470, DOI 10.1038/ncb1800, DOI	2008	8.46	**2010**	2012	
Taylor DD, 2008, GYNECOL ONCOL, V110, P13, DOI 10.1016/j.ygyno.2008.04.033, DOI	2008	6.58	**2010**	2013	
Nilsson J, 2009, BRIT J CANCER, V100, P1603, DOI 10.1038/sj.bjc.6605058, DOI	2009	9.73	**2011**	2014	
Bryant RJ, 2012, BRIT J CANCER, V106, P768, DOI 10.1038/bjc.2011.595, DOI	2012	8.55	**2013**	2016	
Peinado H, 2012, NAT MED, V18, P883, DOI 10.1038/nm.2753, DOI	2012	37.07	**2014**	2017	
Raposo G, 2013, J CELL BIOL, V200, P373, DOI 10.1083/jcb.201211138, DOI	2013	36.04	**2015**	2018	
Hoshino A, 2015, NATURE, V527, P329, DOI 10.1038/nature15756, DOI	2015	28.26	**2018**	2019	
Thery C, 2018, J EXTRACELL VESICLES, V7, P0, DOI 10.1080/20013078.2018.1535750, DOI	2018	55.11	**2020**	2022	

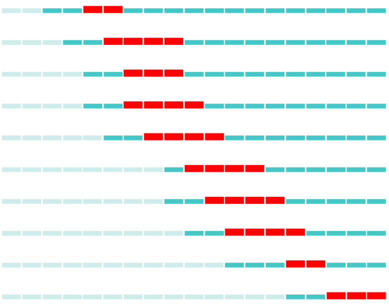

Fig. S4 Top 10 references with the strongest citation bursts generated by CiteSpace software

Table S1 The Top 10 affiliations that contributed to the research of exosomes in prostate cancer

Institutions	Country	Counts	Percentage	Total citation	Average citation per paper	H-index
University of California System	USA	35	3.52	7040	201.14	20
Harvard University	USA	31	3.12	7964	256.9	24
Harvard Medical School	USA	26	2.61	7654	294.38	22
University of Texas System	USA	23	2.31	7011	304.83	18
Shanghai Jiao Tong University	China	19	1.91	848	44.63	13
University of Oslo	Norway	19	1.91	8428	443.58	16
Fudan University	China	18	1.81	1184	65.78	11
Consiglio Nazionale Delle Ricerche-CNR	Italy	17	1.71	5138	302.24	10
University of Toronto	Canada	16	1.61	5746	359.13	12
Chinese Academy of Sciences	China	15	1.51	5325	355	9

Table S2 Top 10 authors and co-cited authors related to exosomes in prostate cancer

Rank	Author	Counts	Total Citations	H-index	TLS	Co-Cited Author	Total Citations	TLS
1	Alicia Llorente	17	8425	15	44	Clotilde Thery	399	16187
2	Gagan Deep	13	734	12	5	Hadi Valadi	244	9931
3	Stefano Fais	11	720	10	32	Hector Peinado	227	10003
4	Mariantonia Logozzi	11	720	10	32	Graça Raposo	217	9749
5	Kirsten Sandvig	11	2123	11	23	Douglas D Taylor	215	11082
6	Lucia R Languino	11	5203	9	31	Sonia A Melo	185	8611
7	Takahiro Ochiya	11	6870	11	23	Johan Skog	185	7798
8	Juan M Falcón-Pérez	11	5243	10	33	Mariantonia Logozzi	180	8794
9	Guido Jenster	10	5433	10	18	Rebecca L Siegel	172	3933
10	Dolores Di Vizio	10	5565	8	28	Marina Colombo	168	7329

TLS, total link strength

Table S3 Top 10 journals and co-cited journals related to exosomes in prostate cancer

Rank	Journals	Counts	IF (2022)	JCR (2022)	H-index	Total Citations	TLS	Co-cited journals	IF (2022)	JCR (2022)	Total Citations	TLS
1	Cancers	40	5.2	Q2	16	1048	256	Plos One	3.7	Q2	2304	268001
2	International Journal of Molecular Sciences	36	5.6	Q1	18	1148	251	Cancer Research	11.2	Q1	2300	250790
3	Oncotarget	28	5.168	Q1	27	2362	318	Journal of Extracellular Vesicles	16	Q1	1930	222468
4	Journal of Extracellular Vesicles	27	16	Q1	19	6237	238	Oncotarget	5.168	Q1	1756	203105
5	Scientific Reports	25	4.6	Q2	15	1047	145	Proceedings of the National Academy of Sciences of the United States of America	11.1	Q1	1585	180589
6	Plos One	22	3.7	Q2	16	1902	246	Scientific Reports	4.6	Q2	1360	172933
7	Prostate	20	2.8	Q2	17	1274	196	Journal of Biological Chemistry	4.8	Q2	1298	153634
8	Frontiers in Oncology	16	4.7	Q2	6	193	183	Nature	64.8	Q1	1169	135393
9	Molecular Cancer	14	37.3	Q1	14	2246	164	Nature Cell Biology	21.3	Q1	1164	133897
10	Cancer Letters	13	9.7	Q1	10	760	105	Cell	64.5	Q1	1018	113499

TLS, total link strength

Table S4 Top 10 cited papers concerning the research of exosomes in prostate cancer

Rank	Title	Journal	Year	First Author	Citations
1	Minimal information for studies of extracellular vesicles 2018 (MISEV2018): a position statement of the International Society for Extracellular Vesicles and update of the MISEV2014 guidelines	Journal of Extracellular Vesicles	2018	Clotilde Théry	4729
2	Secretory mechanisms and intercellular transfer of microRNAs in living cells	Journal of Biological Chemistry	2010	Nobuyoshi Kosaka	1457
3	Current knowledge on exosome biogenesis and release	Cellular and Molecular Life Sciences	2018	Nina Pettersen Hessvik	1327
4	Extracellular Vesicles in Cancer: Cell-to-Cell Mediators of Metastasis	Cancer Cell	2016	Annette Becker, Basant Kumar Thakur	1040
5	Extracellular vesicles in cancer - implications for future improvements in cancer care	Nature Reviews Clinical Oncology	2018	Rong Xu	796
6	Characterization of human plasma-derived exosomal RNAs by deep sequencing	BMC Genomics	2013	Xiaoyi Huang	719
7	Suppression of Exosomal PD-L1 Induces Systemic Anti-tumor Immunity and Memory	Cell	2019	Mauro Poggio	649
8	Exosomes: composition, biogenesis, and mechanisms in cancer metastasis and drug resistance	Molecular Cancer	2019	Ladan Mashouri	616
9	Lipids in exosomes: Current knowledge and the way forward	Progress in Lipid Research	2017	Tore Skotland	590
10	Prostate cancer-derived urine exosomes: a novel approach to biomarkers for prostate cancer	British Journal of Cancer	2009	J Nilsson	563

Table S5 Top 10 co-cited references on research of exosomes in prostate cancer

Title	First author	Jounal	Year	Citations
Exosome-mediated transfer of mRNAs and microRNAs is a novel mechanism of genetic exchange between cells	Hadi Valadi	Nature Cell Biology	2007	244
Glioblastoma microvesicles transport RNA and proteins that promote tumour growth and provide diagnostic biomarkers	Johan Skog	Nature Cell Biology	2008	184
Melanoma exosomes educate bone marrow progenitor cells toward a pro-metastatic phenotype through MET	Héctor Peinado	Nature Medicine	2012	174
Extracellular vesicles: exosomes, microvesicles, and friends	Graça Raposo	Journal of Cell Biology	2013	144
Tumour exosome integrins determine organotropic metastasis	Ayuko Hoshino	Nature	2015	139
Cancer statistics, 2022	Rebecca L Siegel	Ca-A Cancer Journal for Clinicians	2022	135
Prostate cancer-derived urine exosomes: a novel approach to biomarkers for prostate cancer	Jonas Nilsson	British Journal of Cancer	2009	130
Biogenesis, secretion, and intercellular interactions of exosomes and other extracellular vesicles	Marina Colombo	Annual Review of Cell and Developmental Biology	2014	124
Minimal information for studies of extracellular vesicles 2018 (MISEV2018): a position statement of the International Society for Extracellular Vesicles and update of the MISEV2014 guidelines	Clotilde Théry	Journal of Extracellular Vesicles	2018	121
Glypican-1 identifies cancer exosomes and detects early pancreatic cancer	Sonia A Melo	Nature	2015	120
